# On the Temporal Evolution of Key Hemofilter Parameters—In Vitro Study under Co-Current Flow

**DOI:** 10.3390/membranes14090200

**Published:** 2024-09-21

**Authors:** Anastasios J. Karabelas, Alexandra Moschona, Konstantinos Merenidis

**Affiliations:** Chemical Process and Energy Resources Institute, Centre for Research and Technology—Hellas, Thermi, 57001 Thessaloniki, Greece; alexmoschona@certh.gr (A.M.); kostasmere@gmail.com (K.M.)

**Keywords:** hemocatharsis parameters, co-current flow, ultrafiltration coefficient, permeability, sieving coefficient, albumin losses

## Abstract

Effective permeability K_P_, the ultrafiltration coefficient (K_UF_), the sieving coefficient (SC), and the loss/permeation of proteins (primarily albumin) are key parameters/specifications characterizing hemofilter (HF) performance. However, there are uncertainties regarding their determination. This work aims (a) to demonstrate that the co-current flow (of blood and dialysate) can lead to beneficial unidirectional filtration (from blood/plasma to dialysate) under a fairly uniform local trans-membrane pressure (TMP), unlike the presently employed counter-current flow; (b) to study the temporal evolution of key HF performance parameters under co-current flow, particularly during the important early stage of hemocatharsis (HC). Experiments with human plasma and BSA solutions in co-current flow mode (for which a fluid mechanical model is developed) show a fairly uniform local/axial TMP, which also improves the local/axial uniformity of protein membrane fouling, particularly under (currently favored) high convective flux operation. Due to incipient membrane fouling, a significant temporal variability/decline in the effective K_P_ is observed, and, in turn, of other parameters (i.e., the Kuf, SC, and permeation/mass flux M_m_ for albumin and total proteins). A satisfactory correlation of the albumin/protein mass flux M_m_ with permeability K_P_ is obtained, indicating strong inter-dependence. In conclusion, co-current flow, allowing for a fair local TMP axial uniformity, enables the acquisition of accurate/representative data on the evolution of HF parameters, facilitating their interpretation and correlation. The new results provide a basis for exploring the clinical application of the co-current flow.

## 1. Introduction

Since the early days of hemodialysis, which is based on the diffusion of uremic toxins through the membrane, the counter-current flow of blood in fiber lumen versus dialysate in the shell side has been employed in clinical practice to treat End-Stage Renal Disease (ESRD) patients. However, in recent decades, it has been demonstrated (e.g., [1,2]) that other employed hemocatharsis modes (i.e., high-flux hemodialysis or expanded hemodialysis—HDX and hemodiafiltration—HDF), involving significant fluid/plasma convection to dialysate, improve the removal of uremic toxins from ESRD patients; nevertheless, they introduce complications regarding the determination of hemocatharsis performance parameters, which are briefly outlined as follows.

For convenience, the generic acronyms HF (hemofilter) and HC (hemocatharsis) will be used to designate, respectively, the membrane module and the various treatment modes. Key standardized parameters, employed to characterize HF performance, include the ultrafiltration coefficient K_UF_ and the sieving coefficient SC, defined [3] as follows:

K*_UF_* = Q*_UF_*/TMP (1)

SC = 2 C*_UF_*/[C*_fo_* + C*_f_*_1_] (2)

where Q*_UF_* and TMP are the ultrafiltrate rate (mL/min) and the effective trans-membrane pressure, accounting for osmotic pressure difference; C*_UF_*, C*_fo_,* and C*_f_*_1_, which designate the specific species concentration in the ultrafiltrate, fiber/lumen inlet, and fiber/lumen outlet streams, respectively. For the determination of both K*_UF_* and SC, in vitro, standard methods (i.e., ISO, 8637-1 [3]) are commonly employed, involving a “closed system” [3], i.e., without a dialysate feed, only an ultrafiltrate outlet, and a blood-side inlet/outlet flow, using either blood or plasma. Under such conditions, there is a unidirectional trans-membrane flow (i.e., ultrafiltration) from the blood to the dialysate side. Typical K_UF_ and SC *single* values are commonly reported for commercial HF (e.g., [4]), which are obtained under the specific blood-side feed flow rate Q_bin_. It should be also noted that K_UF_ is proportional to the effective hydraulic permeability K_P_, as follows:

K_P_ = K_UF_/S = Q_UF_/[TMP·S] (3)

for the HF membrane surface area S, m^2^. Additionally, this K_P_ definition holds only if there is a unidirectional trans-membrane flow from the blood to the dialysate side.

However, for the following two reasons, the aforementioned standardized/reported single K_UF_ and SC numerical values are of dubious usefulness for the characterization of the HF performance under the actually prevailing clinical conditions: (i) As is well known (e.g., [1,2]), with the currently favored high-flux membranes, particularly in the expanded hemodialysis (HDX) mode, the externally controlled ultrafiltration rate Q_UF_ is not unidirectional. In fact, Q_UF_ is the net value of the “forward filtration” (blood- to dialysate-side) rate in the front part, minus the “back-filtration” rate (from the dialysate to the blood side) in the rear part of the HF module [1,2]. This bidirectionality of the flow, which is different from the unidirectional flow mode of the ISO standard [3], employed for determining both the K_UF_ and SC values, creates problems in clinical data treatment and interpretation (e.g., [5,6,7,8]). Moreover, under such bidirectional flow conditions, the K_UF_ values have no direct quantitative relation with the effective permeability K_P_. (ii) The HF performance is characterized by significant temporal and spatial variability, depending on the implemented HC mode and the imposed flow rate and pressure conditions. For fixed feed flow rates, the temporal HF performance variability (reflected in the varying K_UF_ and SC) is quite strong, particularly in the early stage of the HC treatment/session. Therefore, the commonly reported single K_UF_ and SC “standard” values are merely indicative and of unclear physical significance, as is also evident from recent studies [5,6]. Furthermore, it is well known (e.g., [9,10]) that HF membrane “fouling”, mainly by proteins, is responsible for the gradual reduction in the effective HF membrane permeability and the aforementioned temporal variability. The need to address and resolve the above issues is crucial for improving the HF performance evaluation and motivates this work.

Of particular interest to the present study are results and observations regarding the early stage of membrane–plasma interaction and its impact on membrane permeation and species rejection characteristics. Based on early in vivo studies, Rockel et al. [9] reported that the sieving coefficient of several low-molecular-weight proteins decreases (rather sharply) on membrane exposure to blood, with a tendency to stabilize after approx. 20 min. The authors attributed this tendency to the initial/transient period of formation of a protein layer (or “secondary membrane”) on the HF membrane surfaces. Langsdorf and Zydney [10], working with flat-sheet Cuprophan and PAN membranes, proposed a “two-layer membrane model” involving a fouling layer (comprised of adsorbed plasma proteins) acting as an additional resistance to permeation in series with that of the membrane itself. Boschetti-de-Fierro et al. [11] experimented with dextran sieving to characterize some high-molecular-weight cut-off membranes (before and after exposure to blood). They observed that the sieving coefficients after blood contact exhibited a significant reduction compared to the initial values obtained with the pristine membranes over a seemingly transient period of ~20 min, thus essentially corroborating the earlier results. It should be added that the temporal variability of the HF parameters has not been quantified or modeled for predictive purposes.

**Figure 1:**
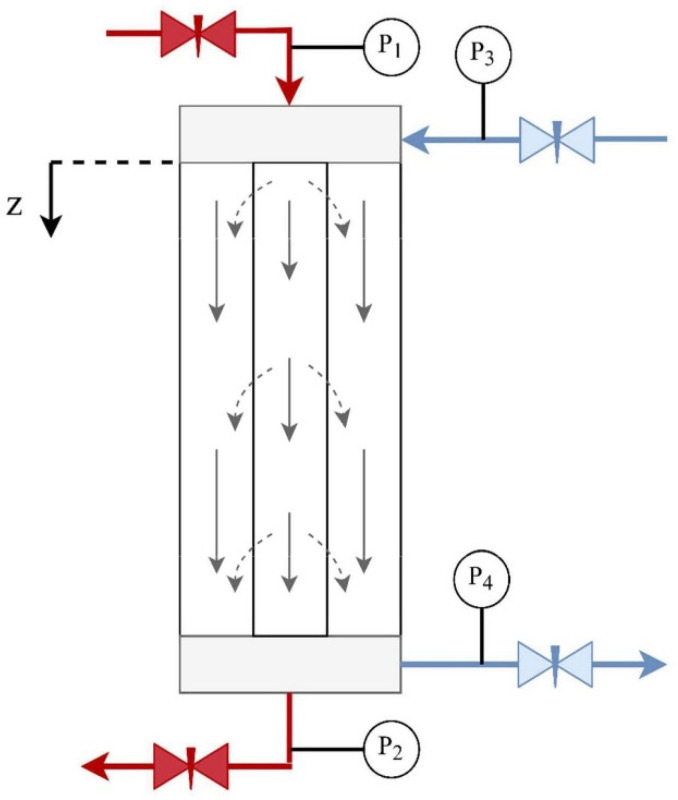
Schematic of the hemofilter in co-current operating mode. P_1_ and P_3_ represent the inlet pressures at the blood side and dialysate side, respectively. The cylindrical active HF section extends between z = 0 and z = L. The shaded regions mark fluid entry/exit end-sections, with the “active” fiber membrane filtration section in-between.

In summary, a significant research priority emerges from the above brief literature review, i.e., the need to determine and correlate, as best as possible, the effective HF membrane permeability K_P_ and related process parameters under sufficiently uniform local TMP and unidirectional (blood- to dialysate-side) flow, focusing on the early stage of the hemocatharsis process. Co-current flow (Figure 1) is employed to implement this approach. As a next step, using the results from such a study, one could proceed to model and predict the HF module performance under various conditions for clinical applications. It should be stressed that the case implemented in this study of co-current (blood- and dialysate-side) flow with significant convection/ultrafiltration has not been dealt with in the HC literature so far. Only very limited work has been reported [12] using conventional hemodialysis, where convection is absent and only species diffusion occurs.

Experiments were performed with a commercial hemofilter with *co-currently* flowing fluids at the lumen and shell sides. Under such conditions, the local/axial variability of the TMP, the fluid permeation rate, foulant deposition, and species rejection tends to be reduced. Therefore, by determining the (inlet and outlet) flow rates and pressures at the two end-surfaces of the cylindrical/active HF section (Figure 1), one can obtain the *mean* parameter values fairly representative of the entire HF. Two types of fluids were employed in this work at the blood side (i.e., human plasma and an aqueous BSA solution as a reference). A theoretical solution to the co-current flow mode of the HF operation, for Newtonian fluids, is also presented in support of the experimental work.

## 2. Methodology—Theoretical Part

### 2.1. Methodology 

The sequence of the main steps in this study is as follows:

i. For the commercial HF [4] employed in this work (Table S1), some fluid mechanical parameters are obtained first [13,14] (as outlined in the Supplement, Section S1, parameters listed in Table S2), aiming to estimate the pressure drop/losses in the HF fluid entry/exit end-sections (Figure 1), thus determining, with improved accuracy, the effective TMP in the active cylindrical membrane section [14].
ii. A mechanistic model is employed to obtain an analytical solution to the co-current flow mode of the HF operation, for Newtonian fluids, in support of the present experimental work and of further modeling studies.
iii. Tests are performed with a BSA solution and commercial HF for the preliminary assessment of the proposed co-current flow protocol.
iv. The main experiments, under co-current flow, are carried out with human plasma, using realistic flow rates prevailing in hemocatharsis, to determine the key HC parameters.
v. Data interpretation of the temporal variation in the HC parameters is presented, including new approaches for their correlation.

### 2.2. Modeling of Co-Current Flow

#### 2.2.1. Analytical Solution

The solution of a mechanistic model is outlined for the co-current operating mode under the steady-flow conditions of Newtonian fluids, such as the aqueous BSA solutions employed in this study. Considering that the fluid mechanical HF parameters (K, $f_{f}$, $f_{s}$, $\zeta_{1},\;\zeta_{2},\;\zeta_{3},\;\zeta_{4})$ are known from the preceding step (Table S2, Supplement), analytical expressions are obtained to determine the axial variation in the main process variables, i.e., the lumen- and shell-side flow rates, and the trans-membrane flux and pressures.

In the following analysis, the fiber lumen and shell side of the HF module are denoted by the subscript “f” and “s”, respectively. The axial flow direction is shown in Figure 1, where z = 0 and z = L correspond (respectively) to the upper (where the blood enters the HF) and lower (where the blood exits the HF) cylindrical surfaces of the active HF section and are designated by the subscripts “0” and “1”, respectively. The mass balance equations for the two sides take the following form:

(4) $$\begin{array}{c} \frac{dQ_{f}}{dz}=-Q_{m} \end{array}$$

(5) $$\begin{array}{c} \frac{dQ_{s}}{dz}=Q_{m} \end{array}$$

(6) $$\begin{array}{c} \frac{dP_{f}}{dz}=-f_{f}Q_{f} \end{array}$$

(7) $$\begin{array}{c} \frac{dP_{s}}{dz}=-f_{s}Q_{s} \end{array}$$

Here, Q_f_ and Q_s_ are the lumen- and shell-side flow rates, respectively, and P_f_ and P_s_ are the corresponding pressures. The quantity Q_m_ (m^2^/s) represents the local trans-membrane flow rate per unit length and is related to the local pressure difference between the two membrane sides, as follows:

(8) $$\begin{array}{c} Q_{m}=K\left(P_{f}-P_{s}\right) \end{array}$$

Assuming that the flow rates at the two inlet positions and at the fiber lumen outlet are known, the boundary conditions are as follows:

(9) $$\begin{array}{c} Q_{f}\left(0\right)=Q_{fo} \end{array}$$

(10) $$\begin{array}{c} Q_{s}\left(0\right)=Q_{so} \end{array}$$

(11) $$\begin{array}{c} Q_{f}\left(L\right)=Q_{f1} \end{array}$$

where K is the membrane permeance.

A pressure boundary condition is not available, and thus only the pressure differences can be calculated. The above system of differential Equations (4)–(7) has an analytical solution. By defining the normalized total friction factor A = {K(f*_f_* + f*_s_*)}^0.5^L (Supplement, Equation (7)), the axial variation in the quantities Q_f_, Q_s_, and Q_m_ can be computed as follows:

(12) $$\begin{array}{c} Q_{f}=-\frac{KL}{A}\left(-\lambda_{1}e^{-A\frac{z}{L}}+\lambda_{2}e^{A\frac{z}{L}}\right)-\frac{\lambda_{3}}{f_{f}} \end{array}$$

(13) $$\begin{array}{c} Q_{s}=\frac{KL}{A}\left(-\lambda_{1}e^{-A\frac{z}{L}}+\lambda_{2}e^{A\frac{z}{L}}\right)-\frac{\lambda_{3}}{f_{s}} \end{array}$$

(14) $$\begin{array}{c} Q_{m}=K\left(\lambda_{1}e^{-A\frac{z}{L}}+\lambda_{2}e^{A\frac{z}{L}}\right) \end{array}$$

The expressions are also obtained for the axial variation in the fiber- and shell-side pressure as follows:

(15) $$\begin{array}{c} P_{f}=\frac{Kf_{f}L^{2}}{A^{2}}\left(\lambda_{1}e^{-A\frac{z}{L}}+\lambda_{2}e^{A\frac{z}{L}}\right)+\lambda_{3}z+\lambda_{4} \end{array}$$

(16) $$\begin{array}{c} P_{s}=-\frac{Kf_{s}L^{2}}{A^{2}}\left(\lambda_{1}e^{-A\frac{z}{L}}+\lambda_{2}e^{A\frac{z}{L}}\right)+\lambda_{3}z+\lambda_{4} \end{array}$$

Finally, substituting the boundary conditions (9)–(11) in Equations (12) and (13) leads to the following expressions for the integration constants λ_1_, λ_2_, and λ_3_:

(17) $$\begin{array}{c} \lambda_{1}=\left[\left(Q_{so}+\frac{\lambda_{3}}{f_{s}}\right)e^{A}+Q_{f1}+\frac{\lambda_{3}}{f_{f}}\right]\frac{A}{KL}{\left(e^{-A}-e^{A}\right)}^{-1} \end{array}$$

(18) $$\begin{array}{c} \lambda_{2}=\lambda_{1}+\left(Q_{so}+\frac{\lambda_{3}}{f_{s}}\right)\frac{A}{KL} \end{array}$$

(19) $$\begin{array}{c} \lambda_{3}=-\left(Q_{fo}+Q_{so}\right){\left(\frac{1}{f_{f}}+\frac{1}{f_{s}}\right)}^{-1} \end{array}$$

An expression for the constant λ_4_ cannot be obtained without a pressure boundary condition; however, if only the pressure differences are of interest, λ_4_ is irrelevant and does not contribute to the solution.

With predetermined fluid mechanical parameters ζ_i_, f_f_, f_s_, and K (Table S2, Supplement) and the known flow rates Q_fo_, Q_so_, and Q_f1_, the calculation procedure is as follows:

i. Parameters λ_3_, λ_1_, and λ_2_ are sequentially computed from Equations (19), (17) and (18), respectively.
ii. The lumen- and shell-side flow rates are obtained from Equations (12) and (13), respectively.
iii. The variation in the local trans-membrane flow rate Q_m_ is obtained from Equation (14).
iv. The lumen- and shell-side pressures are computed from Equations (15) and (16), respectively.

#### 2.2.2. Computation of Ultrafiltration Coefficient K_UF_

The ultrafiltration coefficient K*_UF_* is commonly used in practice to evaluate the performance of HC modules, defined by Equation (1), where the TMP (for negligible osmotic pressure difference) is given by the following expression:

(20) $$\begin{array}{c} TMP=\frac{P_{1}+P_{2}-P_{3}-P_{4}}{2} \end{array}$$

Therefore

(21) $$\begin{array}{c} K_{UF}=\frac{2Q_{UF}}{P_{1}+P_{2}-P_{3}-P_{4}}=\frac{2\left(Q_{fo}-Q_{f1}\right)}{\left(P_{1}-P_{3}\right)+\left(P_{2}-P_{4}\right)} \end{array}$$

For the co-current mode of operation, the following expressions can be obtained for the dominant pressure differences (as outlined in the Supplement):

(22) $$\begin{array}{c} P_{1}-P_{3}=\lambda_{1}+\lambda_{2}+\zeta_{1}Q_{fo}^{2}-\zeta_{3}Q_{so}^{2} \end{array}$$

(23) $$\begin{array}{c} P_{1}-P_{2}=\frac{Kf_{f}L^{2}}{A^{2}}\left[\lambda_{1}\left(1-e^{-A}\right)+\lambda_{2}\left(1-e^{A}\right)\right]-\lambda_{3}L+\zeta_{1}Q_{fo}^{2}+\zeta_{2}Q_{f1}^{2} \end{array}$$

(24) $$\begin{array}{c} P_{2}-P_{4}=\lambda_{1}e^{-A}+\lambda_{2}e^{A}-\zeta_{2}Q_{f1}^{2}+\zeta_{4}{\left(Q_{so}+Q_{fo}-Q_{f1}\right)}^{2} \end{array}$$

By substituting Equations (22) and (24) in Equation (21), the following expression is obtained:

(25) $$\begin{array}{c} K_{UF}=\frac{2\left(Q_{fo}-Q_{f1}\right)}{\lambda_{1}+\lambda_{2}+\zeta_{1}Q_{fo}^{2}-\zeta_{3}Q_{so}^{2}+\lambda_{1}e^{-A}+\lambda_{2}e^{A}-\zeta_{2}Q_{f1}^{2}+\zeta_{4}{\left(Q_{so}+Q_{fo}-Q_{f1}\right)}^{2}} \end{array}$$

The above result shows that the ultrafiltration coefficient is a parameter of the membrane hemofilter, which depends not only on the fluid mechanical parameters ζ_i_, f_f_, f_s_, and K but also on the three flow rates, i.e., Q*_fo_*, Q*_f_*_1_, and Q*_so_*. The same observation was made in the recently proposed counter-current flow modeling of HC modules [14].

## 3. Experimental Part

### 3.1. Materials and Equipment

The commercially available module *Elisio 19H* (Nipro Medical Corporation, Osaka, Japan), comprising the Polynephron™ (polyethersulfone) high-flux hollow fiber membranes, with a 1.9 m^2^ membrane area [4], was employed in all of the HC simulation tests using BSA solutions and human plasma. Its main characteristics are listed in Table S1 (Supplement).

Bovine serum albumin (BSA, Sigma-Aldrich, Darmstadt, Germany) at a concentration of 3 g/L was used as the feed solution at the blood side in some of the tests. The main experiments were conducted using frozen human plasma, kindly provided by the “George Papanikolaou” General Hospital of Thessaloniki. The plasma was received in appropriate packaging after testing for several viruses, such as hepatitis B virus (HBsAg), based on the official guidelines for experimental purposes. All of the experiments were carried out using deionized water at the dialysate side.

The experimental set-up, detailed in previous publications [13,14], was equipped with two feed vessels with a capacity of 5.0 L and 5.5 L for the blood- and dialysate-side fluids, respectively. Two magnetic drive gear pumps (with a flow rate range of 0–1000 mL/min, type MS204, Fluid-O-Tech, Milan, Italy) were employed for both the blood and dialysate feed solutions; they were placed at the top of the module for both solutions (Figure 1). Additionally, four precision pressure transducers (with a range of 0–15 psi, type A-10, Wika, Klingenberg, Germany) were installed at the inlet and outlet at each side of the module. The blood inlet, dialysate inlet, and outlet flow rates were monitored using three flowmeters (101-Flo-Sequate data, McMillan Co., San Francisco, USA). Furthermore, the experimental set-up was equipped with a Programmable Learning Controller, PLC (CMT Series, Weintek, Taipei, Taiwan), for the continuous adjustment, monitoring, and recording of all operating parameters, including pressures and flow rates. Data were continuously monitored and recorded every 30 s.

### 3.2. Experimental Conditions—Test Protocols 

The following three types of experiments are reported here, all performed under co-current flow: (1) a few tests with water for the preliminary assessment of the co-current mode; (2) experiments using an aqueous BSA solution at the blood side in recycling mode; (3) experiments using human plasma at the blood side in the “once-through“ mode. After some type (2) and (3) tests, hemofilter cleaning/washing took place, followed by the measurement of clean water permeability.

In the type (2) tests, 3 L of BSA solution (i.e., 3 g/L in deionized water) was pumped into the hollow fibers at the blood-side inlet (Figure 1), while deionized water was fed co-currently into the shell/dialysate sides. It is noted that this aqueous BSA solution is Newtonian. Two sets of flow rates were tested in this operating mode, i.e., 300/120 mL/min (denoted as experiment “A2”) and 300/360 mL/min (experiment “A4”) for blood/dialysate flow rates, respectively. Intermediate HF cleaning was performed as noted above.

In the type (3) experiments, human plasma was fed at the blood side in the co-current flow mode. However, in these experiments, plasma was pumped “once-through” in the hemodialyzer to maintain a constant feed quality and to closely simulate hemocatharsis conditions. In all of these tests, the flow rates were 250 and 200 mL/min for the blood and dialysate sides, respectively, and three new/clean HF modules (designated as E1, F1, and F2) were employed. It should be stressed that the “once-through” flow mode with the plasma was selected because the preliminary tests in the plasma-recirculating mode exhibited a significant TMP pressure increase. The latter was attributed to the increasing protein concentration in the feed, possible coagulation effects, as well as excessive/unrepresentative membrane fouling. It should be also noted that the plasma samples in tests E1, F1 were used ‘as received’; however, the feed to test F2 was pre-filtered to remove some visible small clots of undetermined origin.

The previously described experiments began after removing air from the system and the module by circulating deionized water in both lumen and shell sides. This step was followed by a co-current clean water test (to determine the clean water permeability K_P_) using deionized water at both sides, lasting for 10 min at the same flow rates as the main experiment. It is noted that (at the set flow rates) the data recording (i.e., designated as time zero) started at the moment the lumen side was filled with the BSA solution (or human plasma).

In each test, the samples were collected from the blood-side feed solution and from both the blood- and dialysate-side outlets. Determination of the albumin and total protein concentration in those samples was carried out in Thessaloniki Gen. Hospital “G. Papanikolaou”. Immuno-turbidimetry was used to determine the albumin in the plasma and dialysate samples by employing ALBU2 (Tina-quant Albumin Gen.2, Roche Diagnostics GmbH, Germany). For the total protein concentration in the plasma and dialysate samples, colorimetry (TP2, cobas c, Roche Diagnostics) and nephelometry (TPUC3, cobas c) were employed, respectively.

Two flow modes were employed for cleaning/washing the used HF modules. In the first cleaning mode, deionized water was pumped exclusively into the lumen side of the dialyzer (entry #1, Figure 1), with the two valves at the dialysate side closed. The aim was to dislodge the proteins and other foulants (i.e., the “gel” layer) covering the membrane/lumen inner surface. In the following cleaning mode, deionized water was fed at one dialysate-side inlet (entry #3, Figure 1) and exited from the blood-side outlet (exit #2, Figure 1), whereas the valves at the blood-side inlet and the other dialysate-side outlet (exit #4) were closed. Cleaning with this mode aimed to remove foulants adsorbed in the membrane pores by the pressurized water, permeating from the shell side into the fiber lumen.

## 4. Results

### 4.1. Module Fluid Mechanical Characteristics under Co-Current Flow

The basic fluid mechanical parameter values for the particular type HF (Elisio 19H) used in this study, accounting for pressure losses at the two end-sections, are listed in Table S2. These values are used to correct the four inlet/outlet pressures at the two ends (z = 0, L; Figure 1) of the active HF cylindrical section.

**Figure 2:**
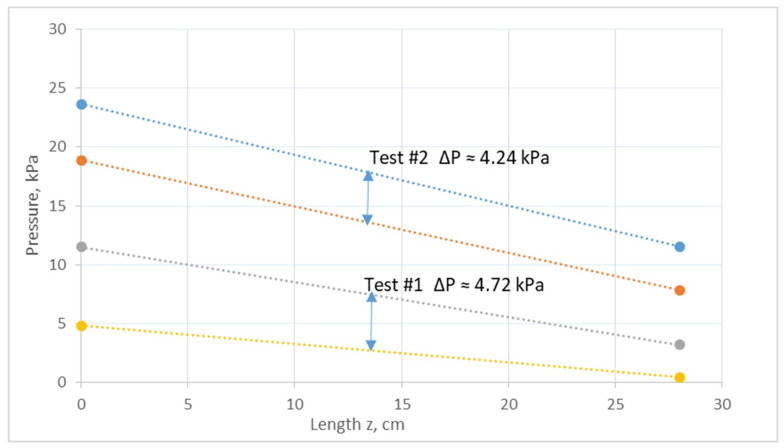
Measured local pressure at the inlet and outlet of the active HF section (Table S3), indicating the fair axial uniformity of the local trans-membrane pressure ΔP. Tests #1 and #2, using water at both sides. Indicative local ΔP marked for each case.

Two preliminary experiments were conducted with water, employing blood-side realistic flow rates, to obtain the salient co-current flow characteristics for Newtonian fluids. The main data of these tests (#1 and #2) are included in Table S3 (Supplement), indicating that for such feed flow rates, the respective Re numbers are of the same magnitude and rather small, as is well known. The measured entry (z = 0) and exit (z = L) pressures, depicted in Figure 2, show that there is a fair uniformity of the local trans-membrane pressure difference (ΔP) in both cases. Moreover, it is evident that the ΔP axial uniformity is significantly improved in test #2, using the same blood-side feed flow rate (Q_fo_) and larger (yet realistic) dialysate-side feed rate (Q_so_) compared to test #1.

**Figure 3:**
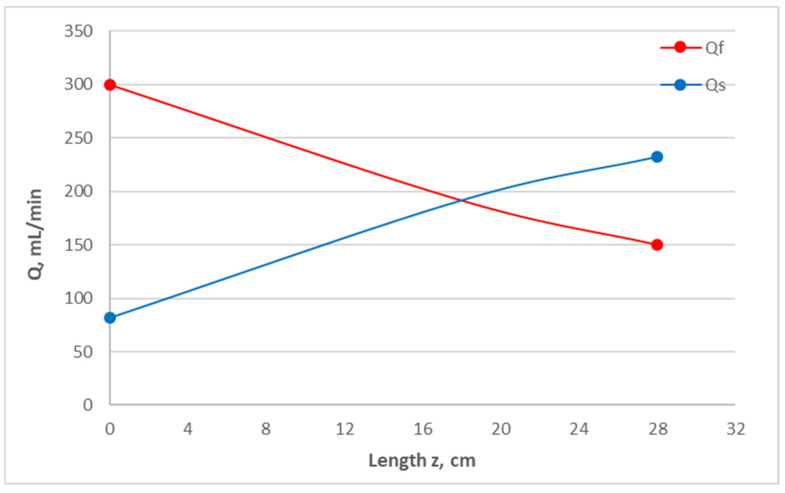
Predicted axial variation of local flow rates Q_f_ and Q_s_ in the lumen and shell side, respectively, indicative of the (modestly variable) local trans-membrane pressure ΔP. Test #1: Q_f_ = 300 mL/min, Q_s_ = 80 mL/min, and Q_UF_ = 150 mL/min.

The above trend, which is due to the difference in the dialysate flow rate Q_so_ (between the two cases), is predictable for Newtonian fluids using the method presented in Section 2.2. For instance, Figure 3 depicts the predicted (for test #1) axial variation in the local flow rates Q_f_ and Q_s_ in the HF lumen and shell side, which are weakly non-linear; therefore, similarly, there is modest variability of the trans-membrane flow rate per unit length Q_m_ (m^2^/s) and of the local trans-membrane pressure difference, as also shown in the pressure data in Figure 2. It should be stressed that, for test #2, the feed flow rates Q_fo_, Q_so_, and Q_UF_ are fairly close to those employed in the clinical HC sessions, while the linearity of the variation in Q_f_ and Q_s_ is significantly improved. These favorable characteristics are a definite attribute of the co-current flow, which are worth further study towards applications in hemocatharsis.

### 4.2. Tests with BSA Solutions

Exploratory experiments with BSA solutions were performed to assess the temporal evolution of the HF key parameters and the overall system performance under co-current flow conditions before the tests with human plasma. The reported tests (A2 and A4) with relatively dilute BSA solutions (i.e., concentration C_bin_ = 3 g/L) were performed in recirculation mode. In Tables S4 and S5 (Supplement) a typical fairly complete listing is provided for the main conditions, data, and computed key parameter values for test A2 and A4. It is noted that test A4 followed test A2 after the cleaning/washing the module to remove the foulants by employing the aforementioned (Section 3.2) cleaning procedure.

**Figure 4:**
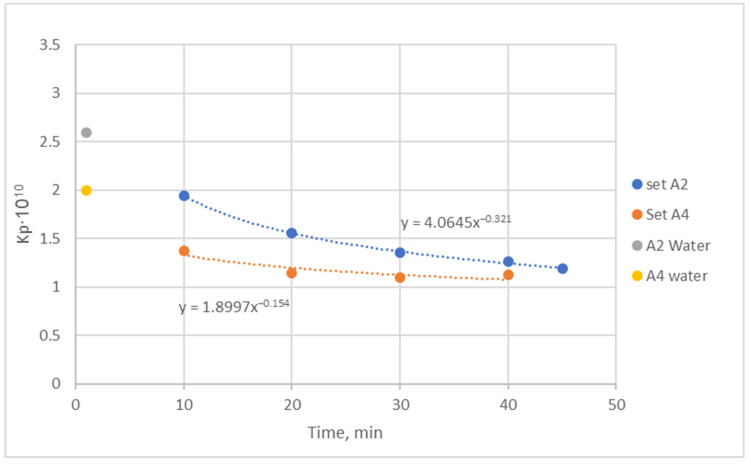
Temporal variation in the effective permeability K_P_. Filtration of the BSA solution; sequential tests A2 and A4 using the same module with intermediate cleaning.

Figure 4 shows very significant temporal variability in the effective permeability K_P_ in these tests. Moreover, higher permeability K_p_ (but with stronger decline) is exhibited by the relatively cleaner membranes in test A2 compared to the subsequent test A4. This trend may be attributed to the inadequacy of the intermediate cleaning process (preceding test A4) to remove all of the BSA/deposits, i.e., apparently, it was impossible to remove the commonly called “irreversible fouling” possibly due to pore blockage/constriction (e.g., [15]). The somewhat greater value of the clean water permeability before test A2, at t ~0 min (i.e., 2.6·10^−10^ m/Pa·s, Table S4), compared to the respective value before test A4 (i.e., 2.1·10^−10^ m/Pa·s) supports this explanation. However, another significant factor likely contributing to the stronger K_P_ decline in test A2 is the greater prevailing ultrafiltration rate, i.e., Q_uf_ ~126 mL/min for test A2 compared to ~60 mL/min for test A4. As is well known from similar studies employing BSA solutions (e.g., [16]), by increasing the permeation flux, membrane fouling is increased, leading to a reduction in its effective permeability.

The difference in the ultrafiltration rate is also most likely responsible for the observed (Table S4) significantly greater BSA leaking into the dialysate side in test A2 during the 45-min tests, i.e., 2.22 g in test A2 as compared to 0.94 g in test A4. However, the estimated (through mass balance calculations, e.g., Tables S4 and S5) additional loss of BSA mass, apparently due to BSA deposition/fouling, appears to be comparable, i.e., 1.52 g and 1.82 g for tests A2 and A4, respectively.

The above comparison of data from tests A2 and A4, showing the different temporal evolution of the permeability K_P_ as well as BSA permeation losses, indicates that the existing *irreversible* membrane fouling before test A4 (likely due to pore constriction and blockage [9,10,15]) plays a dominant role. Furthermore, the significantly reduced (particularly beyond the initial 20 min) temporal variability of K_P_ in test A4 (compared to test A2) suggests that the effect of the fouling layer (i.e., BSA ‘gel’) formation is rather modest and does not substantially affect K_P_.

### 4.3. Tests with Human Plasma

#### 4.3.1. General Flow Characteristics

**Figure 5:**
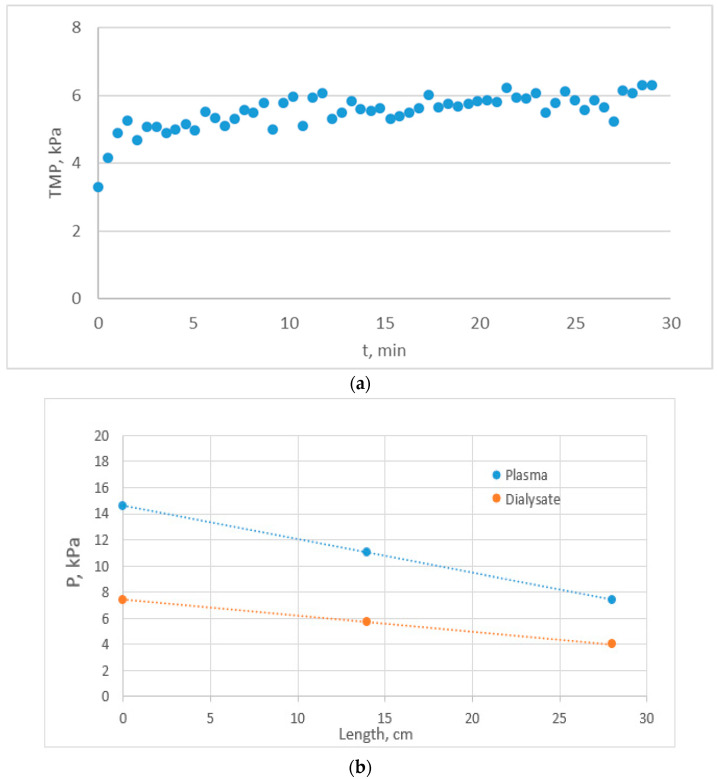
Typical temporal and spatial TMP variation in the co-current flow mode; test E1 with plasma. (**a**) Record of mean TMP temporal variation, showing a modest linear increase after a short initial transient period. (**b**) Typical axial/length-wise variation in the local ΔP, with an estimated mean value ~5 K_P_a at time t = 14 min.

Three simulated hemofiltration tests with human plasma were performed under the same conditions (i.e., Q_Plasma_ = 250 mL/min and Q_Dialysate_ = 200 mL/min) using a new HF module in each test. A fairly complete listing of data and main parameter values (and some mass balance calculations) for tests E1, F1, F2 is presented in Tables S6, S7 and S8, respectively. In two tests (E1 and F1), the *mean TMP* exhibited a small linear temporal increase after a short initial transient period, as shown in Figure 5a. As also observed in the clean water tests (Figure 2), the *local ΔP* along the module axis (Figure 5b) exhibited a relatively small variability, which was maintained throughout the 30 min test. However, in test F2, under the same conditions (Figure S3), the *mean TMP* exhibited (after an initial period of nearly constant TMP) a steadily increasing value. This trend is similar to that observed in clinical studies by Gayrard et al. [17], performed under high-ultrafiltration rates Q_UF_ in the on-line hemodiafiltration (OL-HDF) mode. This may be due to increased protein coagulation phenomena and/or membrane fouling, because the plasma (before this particular test) was pre-filtered to remove visible clots (as noted in Section 3.2); perhaps some remaining clots may have induced coagulation, increased membrane fouling [18] and permeability K_P_ reduction (as shown in Figure S2).

#### 4.3.2. Effective Permeability K_P_ and Ultrafiltration Coefficient K_UF_

**Figure 6:**
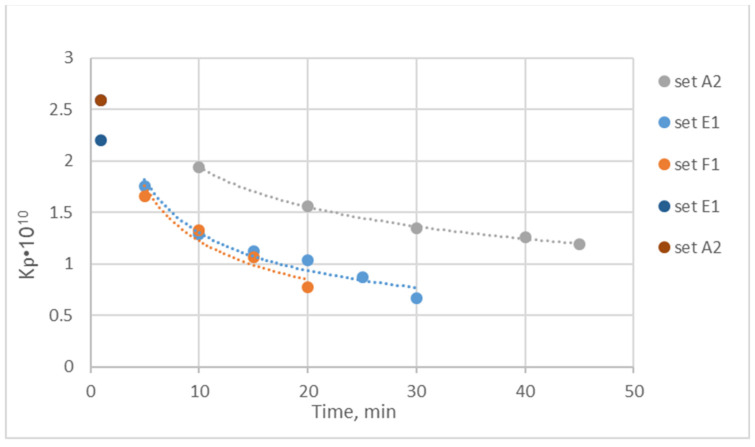
Temporal variation in the effective permeability K_P_ in the simulated hemofiltration with human plasma (tests E1 and F1) under co-current flow exhibiting a significant power law-type decline. Comparison with the BSA solution data (Test A2). Test E1 curve fitting, y = 3.9393x^−0.48^.

Figure 6 depicts the significant initial reduction in the effective permeability K_P_ in the simulated HC with human plasma (tests E1 and F1). It is noted that the measured clean water permeability of the new modules for tests E1, F1 and F2 was K_P_ = 2.60·10^−10^ [m/Pa·s] ± 3%. However, within ~30 min of tests with plasma, the effective K_P_ was reduced to ~25% of that value. This strong continuous decline is well represented by a power law-type function, e.g., y = 3.9393x^−0.48^ for test E1. Significantly, this functional dependence, if extrapolated to a typical hemocatharsis time period of t = 240 min, leads to K_P_ ~2.2·10^−11^ m/Pa·s, which is one order of magnitude smaller than that during the initial 5 min (i.e., K_P_ ~2·10^−10^ m/Pa·s). The reduction in the first 30 min is ~70%, compared to the initial K_P_ value measured with plasma. It should be added that the value reported by the manufacturer [4] of K_UF_ = 76 mL/h/mmHg (using bovine blood [3]) corresponds to K_P_ = 8.34·10^−11^ m/Pa/s, which was obtained in the present tests (Figure 6) at a time of ~25 min.

It is also interesting that, in the present tests, at constant feed flow rates employing plasma (i.e., tests E1 and F1) and the BSA solution (test A2), there is a linear initial temporal variation in both Q_UF_ and TMP due to membrane fouling. However, this variation in Q_UF_ and TMP is of a much different slope regarding both the sign and magnitude, as shown in Figure S1. Therefore, the ratio of these quantities, i.e., K_UF_ = Q_UF_ /TMP and permeability K_P_ = K_UF_/S = Q_UF_ /[TMP·S], is non-linear (Figure 6). Finally, the strong effect of membrane fouling is reflected in the quite different temporal variation in the measured K_P_ in the tests with human plasma and the BSA solution (of a much different consistency), as depicted in Figure 6 and Figure S2 (in semi-log coordinates). Indeed, there appears to be relatively limited fouling in the BSA test A2 compared to significant fouling in plasma tests E1, F1, and F2.

#### 4.3.3. Sieving Coefficient SC and Mass Flux M_m_ of Permeating Species

In addition to the sieving coefficient SC (Equation (2)), the quantities C_UF_ (mean concentration in permeating ultrafiltrate) and M_m_ (the mean permeating species mass flux in mg/min/m^2^) are defined in order to present and interpret the data on the loss of albumin and total proteins (or any other plasma species) permeating/‘leaking’ through the HF membranes. This analysis uses the two measured fixed feed flow rates (Q_fo_ and Q_so_), the mean ultrafiltration rate (Q_UF_), and the albumin (or total protein) concentration in the dialysate at the HF exit (C_D_), as follows:

C_UF_ = {[Q*_so_* + Q_UF_]·C_D_}/Q_UF_ (26)

M_m_ = C_UF_·Q_UF_/S = [Q*_so_* + Q_UF_]·C_D_/S (27)

Also, the instantaneous fiber/blood-side exit concentration C*_f_*_1_ can be estimated by making the fair assumption that it is not affected/reduced by the interaction of blood species with the membrane, as follows:

C*_f_*_1_ = {C*_fo_*·Q*_fo_*}/{Q*_fo_* − Q_UF_} (28)

The important parameter M_m_ (in mg/min/m^2^ of albumin, total proteins, and other species) is introduced in this study, with advantages subsequently discussed.

**Figure 7:**
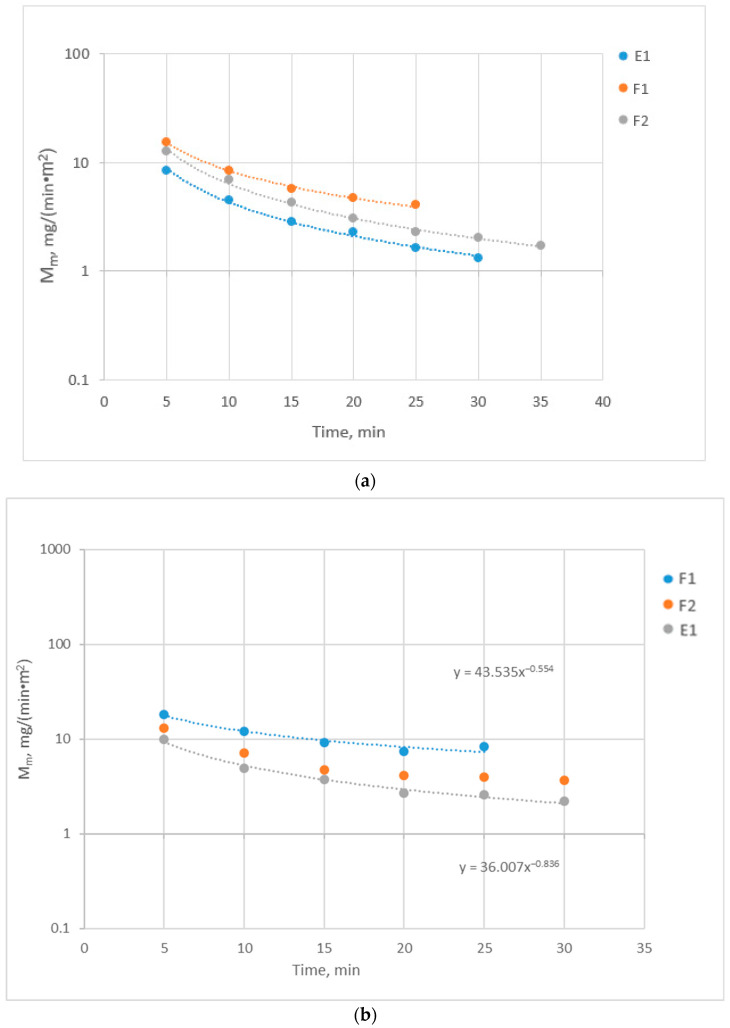
Temporal variation of the permeation mass flux M_m_ [mg/(min·m^2^)] for (**a**) Albumin and (**b**) total proteins, in tests with human plasma under co-current flow, exhibiting rather strong power law-type decline.

Figure 7a,b depicts (in semi-log coordinates) the initial temporal variation in the parameter M_m_ for albumin and total proteins (TPs), respectively, which were computed by employing Equations (26) and (27). These data exhibit an interesting sharp decline (well represented by a power function), which is significantly greater than the decline in the respective effective permeability K_P_ (Figure 6), particularly for albumin. By comparing the magnitude of M_m_ for albumin and the total proteins, it is evident that albumin comprises by far the greatest part of the leaking/permeating total proteins, as is also reflected in the respective measured concentrations of the bulk dialysate C_D_ (e.g., Table S5, Supplement).

**Figure 8:**
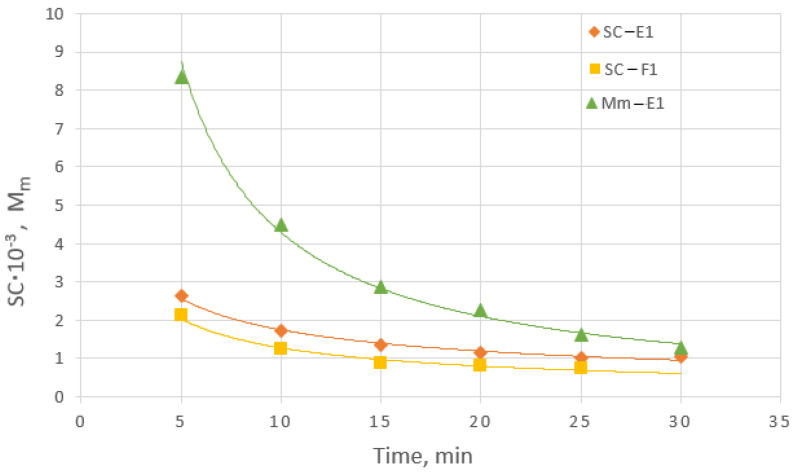
Temporal evolution of the albumin sieving coefficient SC and the permeating albumin mass flux M_m_ for experiments E1 and F1.

In Figure 8, the initial temporal variation in the sieving coefficient (SC) for albumin is presented and compared with respective data on the permeation mass flux M_m_ variation (from Figure 7a). It is interesting that, despite the qualitative similarity of the two types of data, there is much greater initial variation in the mass flux M_m_ compared to the respective SC values. As subsequently discussed, this very pronounced M_m_ variability affords advantages in the presentation and correlation of the mass of “leaking” protein/albumin, as well as of other permeating species during the HC. It should be added that the magnitude of the plotted SC values in Figure 8 is in accord with the SC value (SC < 0.002) for albumin reported by the HF manufacturer [4] (Table S1).

## 5. Discussion

This study has demonstrated that the co-current flow of blood/plasma and dialysate leads to relatively uniform (axially) local TMP, thus ensuring a unidirectional ultrafiltration flow along the entire HF under conditions representative of those prevailing in clinical practice. Since the local TMP is a driving force for fluid/plasma permeation and protein deposition (e.g., [15,16]), such TMP spatial uniformity tends to reduce the axial variation in membrane fouling, which similarly affects the effective permeability K_P_ of the HF, thus benefiting its overall performance. It should be stressed that the observed *temporal* TMP variability is also rather small in typical tests with plasma (Figure 5a). These advantages of the co-current flow compared to the presently practiced counter-current flow facilitate the study of the evolution of key HC parameters and the acquisition of accurate data, particularly under the presently favored high-convection HC modes [1,2]. Therefore, co-current flow merits particular attention in future studies and the further investigation of its application in clinical practice.

To interpret the present data and relate the HF effective permeability K_P_ to membrane fouling, the following general form of the Darcy law is invoked, applicable to *unidirectional transmembrane flow:*

J = [ΔP − Δπ]/[μ(R_m_ + R_c_)] (29)

Here, the quantity J = Q_UF_/S is the mean fluid permeation flux (in mL/min/m^2^), S (m^2^) is the HF effective membrane area, μ is the fluid viscosity, and [ΔP − Δπ] is the mean/effective trans-membrane pressure accounting for the osmotic pressure difference. The total resistance to permeation (R_m_ + R_c_) is comprised of the membrane resistance R_m_ and an additional resistance R_c_, often called “*secondary*” or cake/gel resistance [9,10] due to fouling/deposit formation. However, the clean/unused membrane resistance R_m_ is also increased due to the mechanisms of *pore constriction* and *pore blockage* (e.g., [19,20]) by tightly adsorbed organic species, i.e., by proteins in the case of hemocatharsis [18,21]. It should be noted that these resistances are related to the directly measured quantities in this study, K_p_ and K_UF_, as follows:

K_P_ = 1/[μ(Rm + Rc)] = J/[ΔP − Δπ] = Q_UF_/[S(ΔP − Δπ)] = K_UF_/S (30)

The observed in tests with human plasma (Figure 6) showing a rather strong decline in the effective permeability K_P_ (during the initial ~30 min) is most likely due to the mechanisms of *pore constriction* and *pore blockage*, which tends to increase the membrane resistance R_m_, as also demonstrated in previous studies [15,19,20]. A protein ‘gel’/cake-layer formation (represented by R_c_) possibly follows, caused by larger proteins and possible agglomerates depositing on the initially adsorbed proteins (e.g., [22]). The observed rather small decline of total resistance (R_m_ + R_c_), beyond the initial (~30 min) period, may indicate that pore constriction/blockage is no more effective and that the gel/cake layer is relatively porous. In support of this interpretation is also the small reduction in the resistance (R_m_ + R_c_) beyond an initial period, exhibited by the BSA solution data (Figure 4), in particular with a reused membrane (test A4) that has apparently suffered irreversible fouling due to pore constriction and blockage. However, additional detailed, well-focused experiments are required to clarify and quantify the effect of the deposited protein mass on the HF permeability and to facilitate modeling.

**Figure 9:**
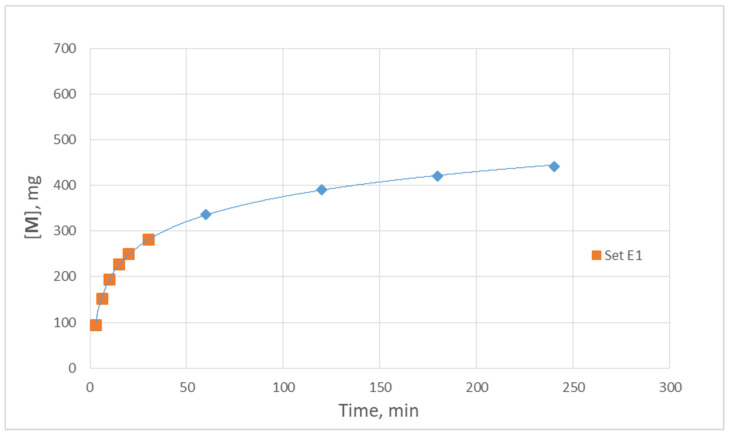
Projected temporal variation of the cumulative albumin loss [M] mg over a 240 min period; in vitro plasma filtration in experiment E1. The albumin mass lost/leaking during the initial 30 min appears to be more than 50% of the total amount lost over a 240 min period. Curve fitting: y = 79.06·ln(x) + 11.32.

The proposed here, for the first time, key parameter M_m_ (permeation mass flux of particular species, e.g., albumin) is considered very useful for direct/facile computation of the total/cumulative mass [M] of species leaking in the dialysate (e.g., albumin [23]) as well as for future modeling studies. A typical realistic example is provided here using the data on M_m_ depicted in Figure 7. For instance, the data on albumin permeation from test E1 show that the temporal M_m_ variation is very well represented by a power function, i.e., M_m_ = 45.897t^−1.03^. Making the fair assumption that this function holds for the entire 4-h period of an HC session, one can readily predict the cumulative (or total) albumin loss to permeate [M] at various times using a simple integration, as shown in Figure 9. It should be noted that the trend as well as the magnitude of these albumin loss projections are in general accord with similar projections by Zawada et al. [24]. However, in that study [24], the less-sensitive sieving coefficient (SC) data were employed, as well as other approximations commonly made for such predictions, e.g., [25].

Regarding the SC data, which are widely used as a performance index (with significant uncertainties in clinical practice, e.g., [7]), the following remarks can be made in comparison with the respective mass flux M_m_ values:

i. An inherent weakness in the definition of the SC is due to the quite small magnitude of the numerator (the dialysate concentration) compared to the denominator (blood concentrations). This leads to the greatly reduced sensitivity of the SC (and increased error margins), thus rendering the SC inappropriate for predictive purposes.
ii. The SC can be reliably determined in vivo *only under unidirectional* ultrafiltration flow from blood to dialysate. Therefore, in HC modes, such as expanded hemodialysis [1], involving both ultrafiltration and back-filtration, the true value of the ultrafiltrate concentration C_UF_ (and therefore SC) cannot be determined.

**Figure 10:**
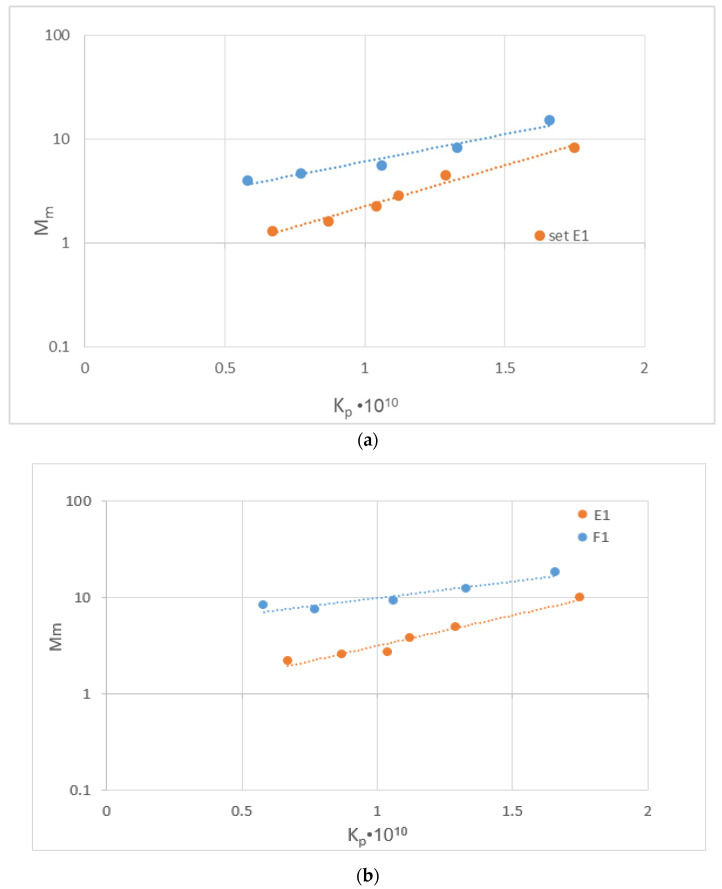
Correlation of species permeation mass flux Mm with effective permeability K_P_ in the simulated hemofiltration with human plasma under co-current flow (tests E1 and F1), exhibiting rather strong (exponential-type) dependence. (**a**) Albumin; (**b**) Total proteins.

**Figure 11:**
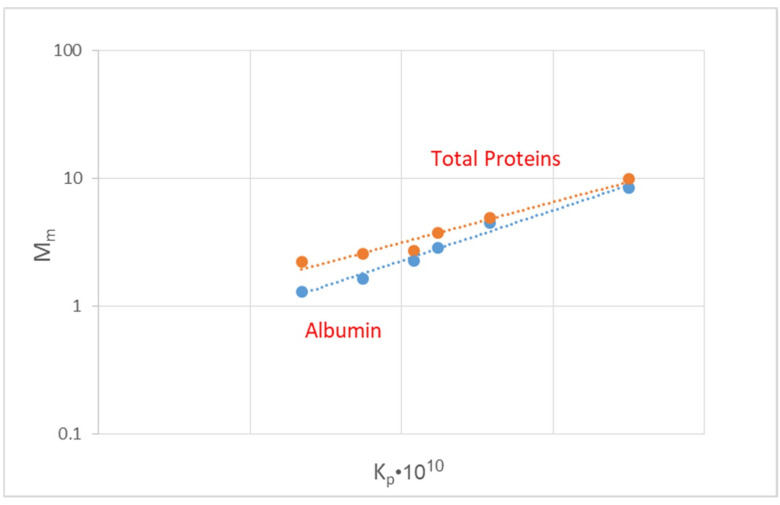
Comparison of the albumin and total protein mass flux M_m_ dependence on the effective permeability K_P_ in the early stage of the simulated hemofiltration with human plasma (test E1). Data fitting: *total proteins* (upper line), y = 0.7266e^1.4614x^; *albumin,* y = 0.3685e^1.8151x^.

The new data (i.e., Figure 6 and Figure 7) suggest that the parameter M_m_, particularly for the middle MW species (such as albumin and some other proteins) depends on the effective permeability K_P_, exhibiting a qualitatively similar temporal evolution. Considering that it is relatively easy to quantify the temporal variability of K_P_, a correlation of M_m_ with K_p_ would be useful. Indeed, as shown in Figure 10a,b, the data from tests E1 and F1 with plasma under co-current flow exhibit a rather strong (exponential-type) dependence of M_m_ on K_p_ for both albumin and total proteins. However, although the trend is qualitatively similar between the two tests (E1 and F1) performed under the same conditions, there is some quantitative difference to be clarified in future studies. It should be also added that there is a somewhat weaker dependence of the total protein mass flux M_m_ on M_p_ (compared to that for albumin), as shown in Figure 11, which is possibly due to the fact that the fouled membrane is more permeable by the smaller-size/MW proteins of the human plasma than by the albumin molecules.

## 6. Conclusions

This study, involving realistic in vitro HC experiments with human plasma (supported by tests with BSA solution), demonstrates that the *co-current flow* direction of blood/plasma and dialysate leads to an axially fairly uniform TMP, particularly under the presently favored high-convection HC modes. In turn, the TMP axial uniformity favors spatially uniform membrane fouling, as well as relatively uniform effective hemofilter permeability K_P_ and overall performance. These conditions facilitate the study of the temporal evolution of key HF parameters, as summarized below. Therefore, the co-current blood and dialysate flow mode clearly deserves particular attention and additional testing towards clinical applications.

Accurate/representative data on the temporal evolution of key HC parameters were obtained, focusing on the HF effective permeability K_p_ and the mass flux K_m_ of permeating albumin and total proteins into the dialysate. K_p_ can be readily determined in co-current flow and exhibits significant temporal variability, particularly during the initial HC period of ~30 min.

The parameter K_m_ introduced here (for the first time for the HC) affords the following clear advantages compared to other similar indices, such as the widely used sieving coefficient (SC): (i) it can be easily determined in vivo under unidirectional trans-membrane flow/ultrafiltration; (ii) it is physically sound (also accounting for the membrane surface area) and is more sensitive than the SC; (iii) it facilitates the accurate determination of the cumulative permeating mass [M] of specific species/solutes, unlike the presently used approximate methods; (iv) it can be readily correlated with other key parameters, particularly the effective K_p_. Such correlations are expected to facilitate modeling and further research towards improved HC clinical applications.

## Data Availability

The original contributions presented in the study are included in the article/Supplementary Material, further inquiries can be directed to the corresponding author.

## Supplementary Materials

The following supporting information can be downloaded at: https://www.mdpi.com/article/10.3390/membranes14090200/s1, Figure S1: Initial temporal variation of TMP (a) and Q_UF_ (b) in co-current flow, employing plasma (Tests E1, F1) and BSA solution (Test A2), at constant feed flow rates; Figure S2: Temporal variation of measured K_P_ in human plasma and BSA tests, depicted in semi-log coordinates. The effect of fouling is evident; i.e., significant fouling in plasma tests (E1, F1, F2), relatively limited fouling in BSA Test A2; Figure S3: Test F2 with plasma. Temporal variation of haemofilter (a) inlet/outlet pressures (Figure 1) and (b) TMP, showing significant linear increase of lumen-side pressure (P1) beyond ~7 min. Q_f_ = 250 mL/min, Q_s_ = 200 mL/min; Table S1: Main parameter values of commercial HF Elisio19H, employed in this work; Table S2: Typical fluid mechanical parameter values of HF Elisio19H, for water at both sides, employing the method [13,14] outlined above; Table S3: Main data from tests #1 and #2 under co-current flow, with water at both sides; Table S4: Test A2—Recycling of BSA solution; Table S5: Test A4—Recycling of BSA solution; Table S6: Test E1—Plasma—“Once-through” flow experiment; Table S7: Test F1—Plasma—“Once-through” flow experiment; Table S8: Test F2—Plasma—“Once-through” flow experiment.
