# Supplementary material for: On the Temporal Evolution of Key Hemofilter Parameters—In Vitro Study under Co-Current Flow
Source: Membranes (Basel). 2024 Sep 21;14(9):200. doi: 10.3390/membranes14090200 (PMC11434192; doi:10.3390/membranes14090200)
Supplement: Supplementary file 1 [file membranes-14-00200-s001.zip › membranes-3175678-supplementary.pdf]

SUPPLEMENT

On the temporal evolution of key hemofilter parameters – In vitro study under co-current flow

S1. Determination of fluid-mechanical parameters of HC Module

For the determination of key HC-module fluid-mechanical parameters, a recently developed novel method [1,2] is used. This method enables estimation of four parameters ζ_i associated with pressure-drop in the module-headers, two friction coefficients f_f and f_s for fiber-lumen and shell-side flow as well as the membrane permeance coefficient K. The mechanistic model is described in detail elsewhere [1,2]; here the data fitting procedure is outlined.

First, the experimental pressure-difference versus flow-rate data, taken with specific operating modes, designated as #3 and #4 [1], are fitted by employing the following equations:

$$P_1 - P_4 = C_1 Q + (\zeta_1 + \zeta_4) Q^2 \tag{1}$$

$$P_2 - P_3 = C_2 Q \tag{2}$$

$$P_1 - P_2 = C_3 Q + \zeta_1 Q^2 \tag{3}$$

$$P_3 - P_2 = C_1 Q + (\zeta_2 + \zeta_3) Q^2 \tag{4}$$

$$P_4 - P_1 = C_2 Q \tag{5}$$

$$P_3 - P_4 = C_4 Q + \zeta_3 Q^2 \tag{6}$$

Here pressures P_1, P_2, P_3, P_4 are the dynamic external pressures, obtained from the raw pressure measurements, by accounting for the static pressure encountered in the experimental unit. Parameters C_1 – C_4 are coefficients of the linear terms, to be determined. By fitting the experimental data points to the curves described by Eqs (1) to (6), the parameters ζ_1 – ζ_4 and C_1 – C_3 are computed (coefficient C_4 is not needed).

Subsequently, the parameters f_f, f_s and K are calculated as follows:

$$A = \sqrt{K(f_f + f_s)}L = cosh^{-1}\left(\frac{C_1}{C_2}\right) \tag{7}$$

$$K = \frac{A}{LC_1 tanh(A)} \tag{8}$$

$$f_f = \frac{2e^A}{(e^A - 1)^2}\frac{C_3}{C_2}\left(\frac{A}{L}\right)^2\frac{1}{K} \tag{9}$$

$$f_s = \left[1 - \frac{2e^A}{(e^A - 1)^2}\frac{C_3}{C_2}\right]\left(\frac{A}{L}\right)^2\frac{1}{K} \tag{10}$$

where A is the normalized total friction factor and L the active fiber/module length. The HF effective permeability K_p can be estimated from the permeance coefficient K through:

$$K_p = \frac{K}{\pi N \left(\frac{D_o + D_i}{2}\right)} \tag{11}$$

where N is the number of fibers in the module and D_i, D_o the inner and outer fiber diameter, respectively. Determined parameter values for the *Elisio19H* module are listed in **Table S2.**

Table S1. Main parameter values of commercial HF *Elisio19H*, employed in this work.

S, m^2	L, cm	N	D_o, µm	D_i, µm	K_{UF}, mL/h/mmHg	SC Albumin
1,9	28	10.700	240	200	76 *	0,002 *

* ISO 8637, 2010

Table S2. Typical fluid mechanical parameter values of HF Elisio19H, for water at both sides, employing the method [1,2] outlined above (**Section S1**)

Parameter (units)	Value
K $(m^2/Pa \cdot s)$	$3.66 \cdot 10^{-9}$
f_f $(Pa \cdot s/m^4)$	$2.70 \cdot 10^9$
f_s $(Pa \cdot s/m^4)$	$0.895 \cdot 10^9$
ζ_1 $(Pa \cdot s^2/m^6)$	$1.40 \cdot 10^{14}$
ζ_2 $(Pa \cdot s^2/m^6)$	$1.27 \cdot 10^{14}$
ζ_3 $(Pa \cdot s^2/m^6)$	$0.52 \cdot 10^{14}$
ζ_4 $(Pa \cdot s^2/m^6)$	$1.33 \cdot 10^{14}$

Table S3. Main data from tests #1 and #2 under co-current flow, with water at both sides.

Test number	Q_{fo} mL/min	Q_{so} mL/min	Q_{UF} mL/min	P_1 psi	P_2 psi	P_3 psi	P_4 psi	u_f cm/s	u_s cm/s	Re_{fo}	Re_{so}
#1	300	82	150	1.67	0.47	0.70	0.07	1.48	0.55	2.96	0.94
#2	300	360	68	3.43	1.68	2.74	1.14	1.48	2.39	2.96	4.12

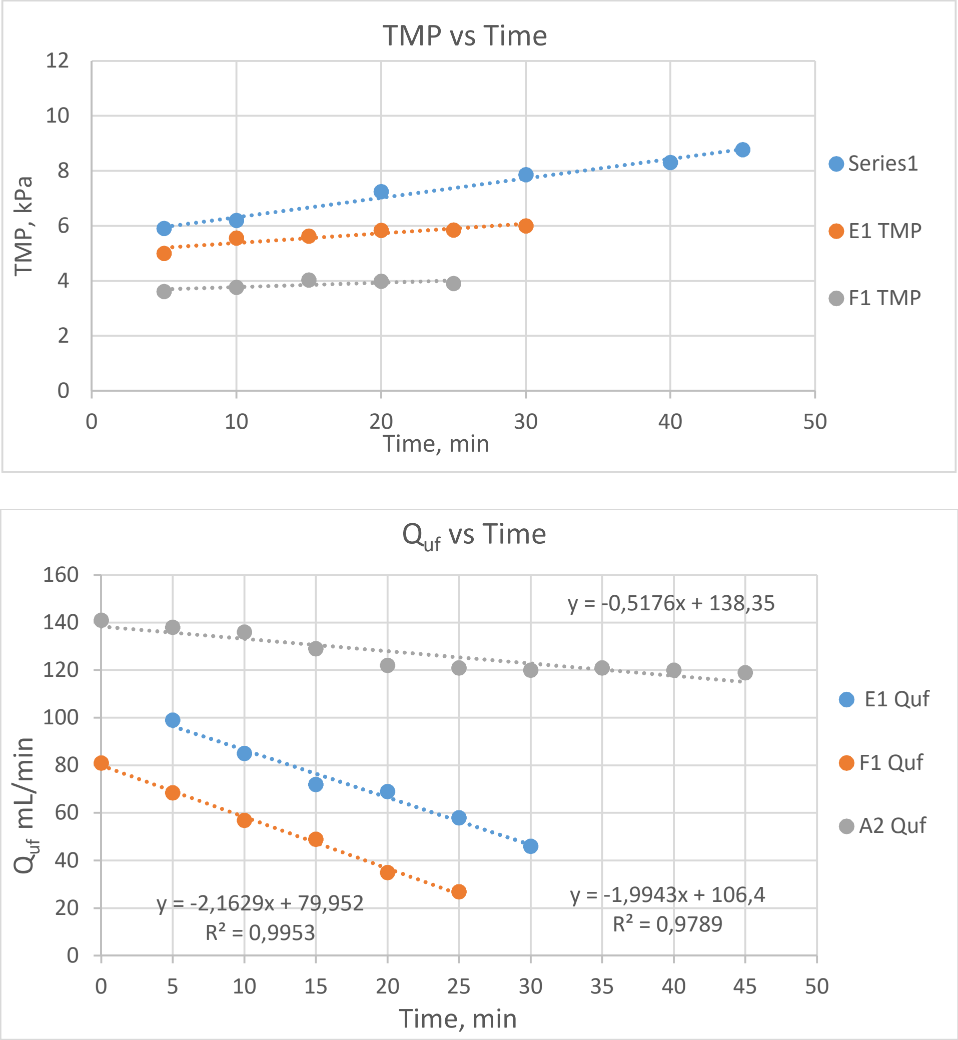

(a)

(b)

Figure S1. Initial temporal variation of TMP (a) and Q_{UF} (b) in co-current flow, employing plasma (Tests E1, F1) and BSA solution (Test A2), at constant feed flow rates.

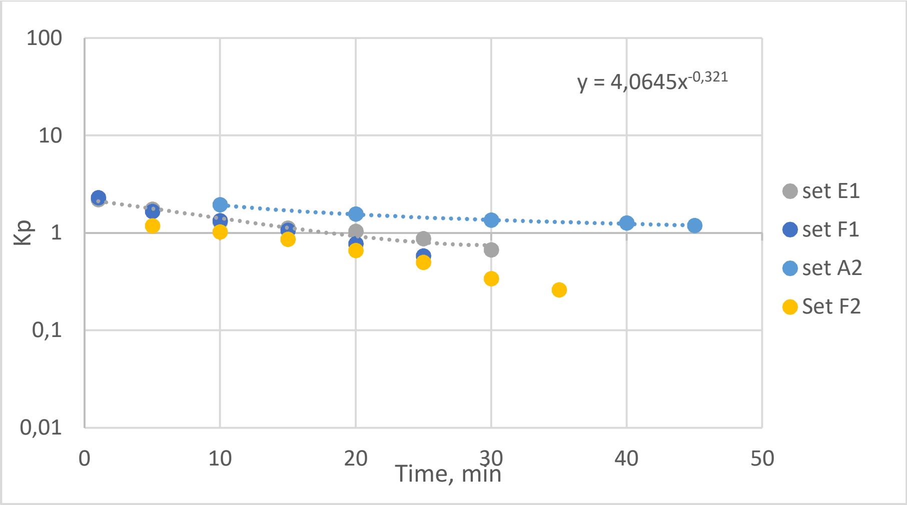

Figure S2. Temporal variation of measured Kp in human plasma and BSA tests, depicted in semi-log coordinates. The effect of fouling is evident; i.e., significant fouling in plasma tests (E1, F1, F2), relatively limited fouling in BSA Test A2.

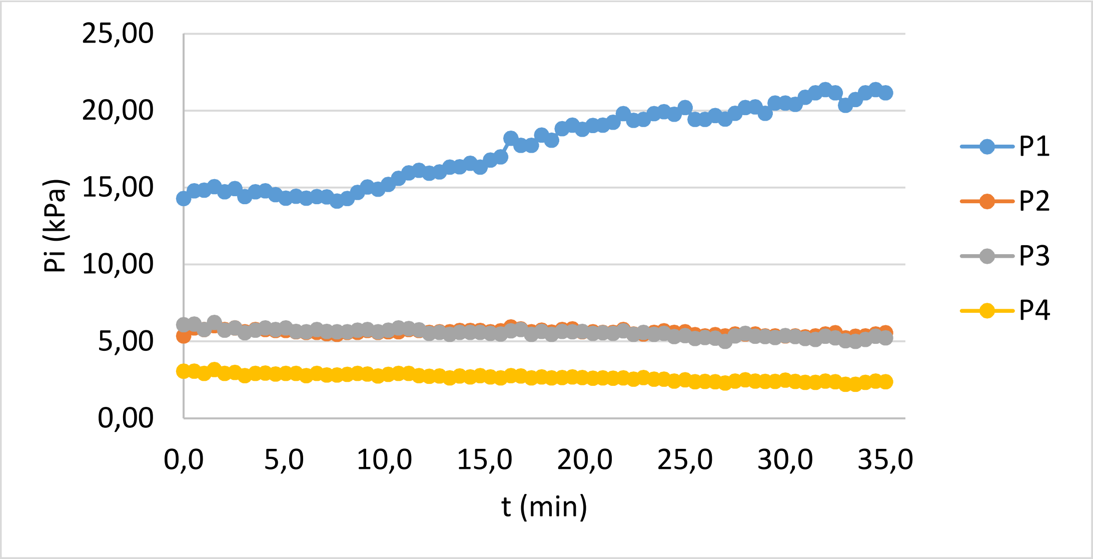

(a)

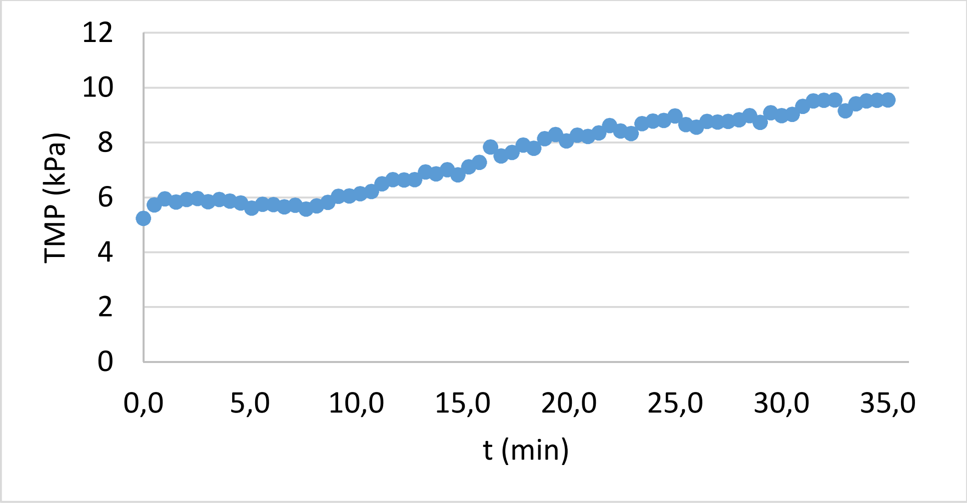

(b)

Figure S3. Test F2 with plasma. Temporal variation of haemofilter a) inlet/outlet pressures (Figure 1) and b) TMP, showing significant linear increase of lumen-side pressure (P_1) beyond ~7 min. Q_f = 250 mL/min, Qs = 200 mL/min

Literature cited

[1] A.J. Karabelas, M. Kostoglou, A. Moschona, D.C. Sioutopoulos, Method development for experimental determination of key fluid-mechanical parameters of haemo-catharsis modules, J. Membr. Sci. 611 (2020) 118353. https://doi.org/10.1016/j.memsci.2020.118353

[2] M. Kostoglou, A. Moschona, A.J. Karabelas, D.C. Sioutopoulos, Implementation and validation of an innovative method for fluid mechanical characterization of haemo-catharsis modules, J. Membr. Sci. 624 (2021) 119117.https://doi.org/10.1016/j.memsci.2021.119117

Table S4. **Test A2 - Recycling of BSA solution**

Q_{BSA} = 300 mL/min, $Q_{Dialysate}$ = 120 mL/min, ***Initial BSA-solution volume V_{bo} = 9 L*** **Temperature: 26.2 °C**

**t** min	***Q_{UF}** mL/min	**Q_{UF} **** mL/min	**TMP** kPa	**$K_{UF}\cdot10^{10}$** m^3/Pa·s	**$K_p\cdot10^{10}$** m/Pa·s	**# C_{BSA} /out,** g/L	[V_{BSA}]*** L
^0	**141**	141	**4,78**	**4,92**	**^2,59**		**9,0**
1		140					
**5**	**138**	**138**	5,90			5,67	8,31
10		**137**	**6,2**	**3,68**	**1,94**	**6,19**	**7,62**
**15**	**136**	**136**				6,71	
20		**129**	**7,24**	**2,97**	**1,56**	**7,23**	**6,26**
**25**	**122**	**122**				7,45	
**30**		**121**	**7,86**	**2,57**	**1,35**	**7,67**	**5,04**
35	**120**	**120**				7,89	
40		**119,5**	**8,3**	**2,40**	**1,26**	**8,10**	**3,84**
**42,5**	119	**119**				**8,31**	
45	119	119	**8,77**	**2,26**	**1,19**		**3,308**
**Mean [Q_{UF}] = 126.5 mL/min**			**End-of-test/Bulk Concentration :**			[7,03]	

* Measured mean Ultrafiltration Rate in each 10 min interval, **Value at each point in time, **V_{BSA}***** Feed volume remaining
# C_{BSA} /out, BSA concentration exiting the module, ^ clean water permeability

Total dialysate volume V_D= 11,092 L (end of experiment), Dialysate final bulk concentration C_D = 0,2 g/L

Total [V_{UF}] =**Total dialysate vol. V_D 11,092 L** (end of exp.) – **5,4 L fed** = **5,692 L** **Final [V_{BSA}] = 9,0 L - 5,692 L = 3,308 L**

Initial BSA mass [9L• 3g/L] = 27g **Final BSA mass [3,308 •7,03]=23,26 g** **BSA leakage in dialysate = [11,092]•[0,2]= 2,22 g**

Total BSA loss= 27 – 23,26 = 3,74g **BSA mass loss due to deposition/fouling = 3,74 – 2,22 = 1,52 g**

Table S5. Test A4 - Recycling of BSA solution

Q_{BSA} = 300 mL/min **$Q_{Dialysate}$ = 360 mL/min** ***Initial volume V_{bo} = 6,39 L*** **Temperature: 26.0 °C**

t (min)	Q_{UF} * (mL/min)	Q_{UF} ** (mL/min)	TMP (kPa)	$K_{UF} \cdot 10^{10}$ (m^3/Pa·s)	$K_p \cdot 10^{10}$ (m/Pa·s)	#C_{BSA} /out g/L	[V_{BSA}] L
0^	68		**2,83**	4,00	**^2,11**	3,0	**6,39**
5	**64**	**64**					
10		**61,5**	**3,94**	**2,60**	**1,37**	**3,61**	**5,75**
15	**59**	**59**					
20		**59,5**	**4,6**	**2,16**	**1,14**	**4,05**	**5,16**
25	**60**	**60**					
30		**59,5**	**4,8**	**2,07**	**1,10**		**4,56**
35	**59**	**59**					
40	59	59	**4,61**	**2,14**	**1,12**	**4,62**	**3,97**
45	[2715] mL		2,95				**[3,705]**
			**End-of-test/Bulk Concentration :**			**[4,42]**	

* Measured mean Ultrafiltration Rate in each 10 min interval, ** Value at each point in time, **V_{BSA}***** Feed volume remaining
# C_{BSA}/out, BSA concentration exiting the module, ^ clean water permeability

Total dialysate volume = 18875 mL (bulk/ end of experiment), Dialysate final bulk concentration C_D = 0,05 g/L Mean Q_{UF} = 60 mL/min

$V_{InitialBSA}$=6,39 L, $V_{FinalBSA}$=3,705 L BSA Mass Initial = 19,17g BSA Mass remaining= [3,705x4,42] = 16,38g

Total BSA loss= 19,17 – 16,38 = 2,76 g BSA leakage in dialysate = [18,875 • 0,05] = 0,94 g

BSA mass loss due to deposition/fouling = 2,76 – 0,94 = 1,82 g

Table S6. <u>Test E1</u> **Plasma – "Once-through" flow experiment**

Q_{Plasma} = 250 mL/min, $Q_{Dialysate}$ = 200 mL/min, ***Initial plasma volume V_{bo} = 8L*** **Temperature: 26 °C**

t min	*Q_{UF} mL/min	**Q_{UF} mL/min	TMP kPa	$K_{UF} \cdot 10^{10}$ m^3/Pa·s	$K_p \cdot 10^{10}$ m/Pa·s	**# C_D mg/L**	## C_D mg/L	**C_{UF} mg/L**	**C_{Bout} g/L**	**SC** Album	#M_m mg/ (min•m^2)	##M_m mg/ (min•m^2)	**V_D^^ L**	**M###** mg
**^0**	77.75		**2,55**		**^2,67**									
~1		~102	**4,0**	**4,25**	**~2,2**									
2,50	102,0					53	63						**1,51**	**> 80,0**
**5,00**		**99,0**	**4.97**	**3.32/150,7**	**1,75**	**53**	**63**	**160**	**76**	**0,0026**	**8,34**	**9,91**		
7,50	96,0					42	48						**1,48**	**62,2**
**10,0**		**85,0**	**5.79**	**2.45/122,9**	**1,29**	**30**	**33**	**100,6**	**69,5**	**0.0017**	**4,49**	**4,93**		
12,5	74,0					25	30						**1,37**	**34,2**
**15,0**		**72,0**	**5.63**	**2.13/102,2**	**1,12**	**20**	**26**	**75,6**	**64,5**	**0.0014**	**2,87**	**3,74**		
17,5	70.0					18	23						**1,35**	**24,3**
**20,0**		**69,0**	**5.84**	**1.97/94,6**	**1,04**	**16**	**19**	**62,4**	**63,4**	**0,0011**	**2,26**	**2,69**		
22,5	68,0					14	19						**1,34**	**18,8**
**25,0**		**58**	**5.85**	**1.65/79,2**	**0,87**	**12**	**19**	**53,4**	**59,8**	**0.0010**	**1,63**	**2,58**		
27,5	48.0					11	18						**1,24**	**13,6**
**30,0**		**46**	**6.0**	**1.28/61,4**	**0,67**	**10**	**17**	**53,5**	**56,3**	**0,0010**	**1,30**	**2,20**		
**Mean [Q_{UF}]= 76.3 mL/min**			**End-of-test/Bulk Concentration:**			**[31]**	**[34]**					**Total :**	**[8,29]**	

***** Measured mean Ultrafiltration Rate in each 5 min interval, **Value at each point in time, **V_D^^** Dialysate volume exiting over a 5min period
#C_D, #M_m and ##C_D, ##M_m for Albumin and Total Proteins in dialysate exiting the module, **M###** Albumin mass lost/leaking over a 5 min period

Total dialysate volume out = 8,29 L (bulk/end of experiment) ^ time t=0, clean water permeability K_{UF} Units: **mL/hr/mmHg**
<u>**Albumin**</u> **total loss/leaking [8,29L · 31mg/L]=** ***257mg*** <u>**Total Proteins**</u> **total loss/leaking [8,29L · 34mg/L]=** ***281,9mg*** **Feed: C_{Bin}=45,9 g/L**

Table S7. Tests F1 Plasma – “Once-through” flow experiment

Q_{Plasma} = 250 mL/min, $Q_{Dialysate}$ = 200 mL/min, *Initial volume* V_{bo} = *6,5 L* Temperature: 37,5 °C

t (min)	[Q_{UF}]* mL/min	Q_{UF} ** (mL/min)	TMP (kPa)	$K_{UF} \cdot 10^{10}$ (m^3/Pa·s)	$K_p \cdot 10^{10}$ (m/Pa·s)	#C_D (mg/L) # Album.	C_{UF} (mg/L) # Alb.	C_{Bout} g/L	SC •10^{-3} Album.	M_m mg/ (min•m^2) Albumin	##C_D (mg/L) Total Pr	M_m mg/ (min•m^2) Total Prot.	V^^ L	M### mg Album.
0^		81.00	2,75	4,9	2,58^									
~ 1		~ 80	~ 3,2	4,4	~2,2									
	77.00					109							1,385	151
5.0		68,5	3.61	3.16/151,7	1,66	109	427,2	59,64	2,12	15,4	130	18,4		
	60.00					85							1,300	110,5
10.0		57,0	3,76	2.53/121,4	1,33	62	279,5	56,09	1,25	8,4	90	12,17		
	54.00					52							1,270	66,0
15.0		49,0	4.03	2.03/97,4	1,07	43	218,5	53,86	0,89	5,64	70	9,17		
	44.00					40							1,220	48,8
20.0		35,0	3.98	1,47/70,6	0,77	38	255,1	50,35	0,81	4,70	60	7,45		
	26.00					36							1,130	40,7
25,0		26,0 ?	3,90	1.11/53,3	0,58	34	295,5	48,33	0,74	4,04	70	8,30		
		[52,0]	[End-of-test/Bulk Concentration] :			[66]					[90]			
												Total :	6,305	417

* Measured mean Ultrafiltration Rate in each 5 min interval, ** Value at each point in time, V_D^^ Dialysate volume exiting over a 5min period

#C_D , ##C_D for Albumin or Total Proteins in dialysate exiting the module, M### Albumin mass lost over a 5 min period K_{UF} Units: **mL/hr/mmHg**

^ clean water permeab. **Total dialysate volume out = 6,305 L** Total Plasma fluid permeating the membranes over the 25 min period = **1,305L.**

Total Albumin loss [6,305· 66mg/L]= *416,1mg* **Total Protein loss [6,305· 90mg/L]= *567,5mg*** **Feed: C_{Bin} = 43,3 g/L**

Note: 417mg Albumin loss determined from additive losses in each 5min time-period

Table S8. Test F2 Plasma – “Once-through” flow experiment

Q_{Plasma} = 250 mL/min, $Q_{Dialysate}$ = 200 mL/min, ***Initial volume V_{bo} = 9L*** **Temperature: 37,2 °C**

t (min)	Q_{UF} * mL/min	Q_{UF} ** mL/min	TMP (kPa)	$K_{UF} \cdot 10^{10}$ (m^3/Pa·s)	$K_p \cdot 10^{10}$ (m/Pa·s)	C_D # (mg/L)	C_D ## (mg/L)	M_m # mg/(min•m^2)	M_m ## mg/(min•m^2)	**V_D* L**	**$M^{###}$ mg**
0*	77.25		**2,68**	**4,8**	**2,53**^						
~1	82	82	**5,24**	2,61	**1,37**						
2,5	**80.00**					87	90			**1,40**	~121,8
5,0		**76**	**5,61**	**2.26/107,5**	**1,19**	**87**	**90**	**12,6**	**13,15**		
7,5	**71.00**					65				**1,36**	88,4
10,00		**70,5**	**6,13**	1.92/**92,2**	**1,01**	**48**	**50**	**6,83**	**7,14**		
12,5	**70.00**					39				**1,35**	52,7
15.00		**67**	**6,83**	1.63/**78,2**	**0,86**	**30**	**34**	**4,22**	**4,78**		
17,5	**64.00**					26				**1,32**	34,3
20.00		**62**	**8,06**	1.28/**61,4**	**0,68**	**22**	**30**	**3,04**	**4,14**		
22,5	**60.00**					19				**1,30**	24,7
25.00		**51**	**8,97**	0.95/**45,6**	**0,50**	**17**	**30**	**2,26**	**3,99**		
27,5	**42.00**					16,5				**1,21**	20,0
30.00		**35**	**8,99**	0.65//**31,1**	**0,34**	**16**	**30**	**2,02**	**3,69**		
32,50	**28.00**					15				**1,14**	17,1
35,0		**28**	**9,56**	0,49/**23,4**	**0,26**	**14**	**15**	**1,70**	**1,80**		
	**2075 mL**		**End-of-test/Bulk Concentration:**			**[44]**	**[50]**				
									**Total :**	**[9,08]**	**~[359]**

* Measured mean Ultrafiltration Rate in each 5 min interval, ** Value at each point in time, **V_D*** Dialysate volume exiting over a 5min period
# C_D /M_m and ## C_D /M_m for Albumin and Total Proteins in dialysate exiting the module, **$M^{###}$** Albumin mass lost/leaking over a 5 min period

Total dialysate volume = 9075 mL (bulk/ end of experiment) ^ clean water permeability K_{UF} Units: **mL/hr/mmHg**

Albumin loss/leaking [9,075L · 44 mg/L]= ***399,3mg*** **Total Protein loss/leaking [9,075L · 50mg/L]=** ***453,8mg***
